# Review of *Trichomonas vaginalis* in Iran, Based on Epidemiological Situation

**Published:** 2018

**Authors:** Mohsen Arbabi, Mahdi Delavari, Zohreh Fakhrieh-Kashan, Hossein Hooshyar

**Affiliations:** - Department of Medical Parasitology, School of Medicine, Kashan University of Medical Sciences, Kashan, Iran

**Keywords:** Epidemiology, General population, Iran, Prevalence, *Trichomonas vaginalis*

## Abstract

Trichomoniasis, which is caused by *Trichomonas vaginalis*, is the most common non-viral sexually transmitted infection (STI) in the world including Iran. There were roughly 250 million new cases all over the world in a year. *T. vaginalis* as an important disease has been associated with HIV (in terms of exposure to sexually transmitted infection, STI) which increases the number of high-risk members, and thus it is an important public health problem. Additionally, this pathogen has been associated with serious health consequences. For instance, it may cause a woman to deliver a low-birth-weight or premature infant, and increase chances of cervical cancer. Because little information is available about the prevalence of *T. vaginalis* infection in Iranian population, this review was carried out to determine the prevalence of *T. vaginalis* among Iranian population. For this systematic review, data about epidemiology of *T. vaginalis* in different parts of Iran with different populations were systematically collected from 1992 to 2017 through the international databases such as PubMed, Scirus, ISI Web of Science, Scopus, EMBASE, Science Direct and Google Scholar and Islamic World Science Citation Center (ISC). National database searching included Iran Medex, Iran Doc, Magiran and Scientific Information Database (SID). A total of 39 clinical and laboratory investigations about the prevalence of Trichomoniasis from different regions of Iran were analyzed. The overall prevalence rate of *T. vaginalis* infection in Iranian population was estimated to be minimally 0.4% and maximally 42%. The present review showed that *T. vaginalis* infection rate is relatively high among the Iranian population. The control strategies, including personal hygienic education, simultaneous couple treatment, the sensitivity of diagnostic methods, appropriate preventive tool (condom) in sexual contacts could lead to the disruption of transmission.

## Introduction

Trichomonas vaginalis is the causative agent of trichomoniasis, a non-ulcerative sexually transmitted disease. *T. vaginalis* is the protozoan parasite infecting the urogenital tract of both females and males (1, 2). It is reported to be 250 million new cases all over the world every year (3). Estimates of prevalence are differences between populations, but the range from 5–74% in women and 5–29% in men is observed (1). Women by the age of 16–53 are at greater risk of infection (4–6). Recently, different studies have shown that *T. vaginalis* has been associated with HIV, which increases the number of high-risk members (7, 8). Additionally, this pathogen has been associated with serious health consequences; including low-birth-weight in pregnant woman or premature infant and increased chances of cervical cancer. Women who are infected can be asymptomatic or have different symptoms, consisting a yellowish-green frothy discharge purities, dysuria, and the strawberry cervix which is recognized by punctuates hemorrhagic lesions. In general, infection is asymptomatic in men, although it can be associated with urethral discharge and dysuria (1–4). For diagnosis of trichomoniasis, different methods have been used, such as, wet mount, culture, Papanicolaou smear, Pap smear, polymerase chain reaction (PCR) and serological tests. Wet mount tests are quick and straightforward. Specialized medium cultures are used for diagnosis, but 2–5 days are required. Also, in some cases, parasites are diagnosed in the Papanicolaou smear. Moreover, the lack of sensitivity and specificity of serological examines is the major limitation for the detection of *T. vaginalis* by indirect serological testing. Recently, new approaches to diagnosis of parasite infections are provided by molecular biological methods. PCR allows the amplification of DNA fragments and diminishes the probability of misdiagnosis. When few trophozoites are recognized in a man’s reproductive organs, it is believable that PCR is useful for the diagnosis of trichomoniasis (6–9). 5′-nitroimidazole family include metronidazole and tinidazole which are the most common drugs for treatment of trichomoniasis (10, 11). Because little information is available about the prevalence of *T. vaginalis* infection in Iranian population, this systematic review was carried out to determine the prevalence of *T. vaginalis* infection among Iranian population from 1992 to 2017 through the electronic databases.

### Literature review of prevalence of trichomoniasis:

For this systematic review, electronic searches in international and national databases and journals were conducted using key words of *Trichomonas vaginalis*, general population, prevalence, epidemiology, and Iran.

These articles had used at least one method (Direct smear, culture, PCR, Pap smear) for epidemiological study in different parts of Iran. Searches were performed through the international databases such as PubMed, Scirus, ISI Web of Science, Scopus, EMBASE, Science Direct, Google Scholar and Islamic World Science Citation Center (ISC). National database searching included Iran Medex, Iran Doc, Magiran and Scientific Information Database (SID). Articles that were published between 1992 and 2017 were reviewed. Among the numerous information sources, relevant studies on *T. vaginalis* infection were identified. Articles related to women with childbearing age who referred to health centers due to pregnancy care and symptoms of vaginitis as well as gynecologic problems and men in different parts of Iran were included. A total of 39 papers were investigated and 3 articles related to women prisoners were excluded from the study. Due to high-risk relationships and inappropriate health conditions, the prevalence of vaginal Trichomoniasis in female prisoners is high and is different from normal rate in the society. Therefore, articles published in the prison population were excluded. For this pathogenic parasitic protozoa, the studies were contradictory and generally of poor quality. At present, different methods have been used for recognizing Trichomoniasis, such as culture, Pap smear, polymerase chain reaction (PCR), and direct smear. Thus, analysis of data was based on the diagnosis of *T. vaginalis* parasite in women who referred to health centers. Results were obtained from different geographical areas classified and analyzed by descriptive statistics.

Women referred to health centers in Sirjan (1992), Kashan (1993), Isfahan (1995) and Tabriz (1998) were infected with *T. vaginalis* (Direct smear: 2.2% and culture: 2.8%), (Culture: 2 0.1%), (Direct smear: 1.49% and culture: 1.92%), (Direct smear: 22.6%), respectively (12–15). Pap smear specimens from women referring to health centers in Yasouj (1999) were 1.9% infected with *T. vaginalis* (16). In 1010 Pap smear specimens (1999) of 9.9% patients referring to Kashan, were infected with *T. vaginalis* (17). Pregnant women referring to health centers in Kashan (2000) and women referring to health centers in Zahedan (2001) and Hamedan (2001) were infected with *T. vaginalis* (Culture: 0.44%), (Direct smear: 4.5% and culture: 5.3%), (Direct smear: negative), respectively (18–20). Result of the direct smear method and culture in pregnant women referring to health centers in Tehran (2002) showed 2.9% of patients were infected with *T. vaginalis* (21). Women referring to health centers in Gorgan (2003), Hamedan (2004) and Orumieh (2004) were infected with *T. vaginalis* (Direct smear and culture: 9%), (Direct smear: 2% and culture: 3%), (Direct smear: 2.4% and culture: 2.6%), respectively (22–24). 63 women with symptoms of vaginitis in Yasouj were surveyed using direct smear and culture methods (2004) and the result showed 19.04% and 42.9% of patients were infected with *T. vaginalis*, respectively (25). In the health center in Robat Karim (2005), patients were tested by direct smear and culture; results showed 1.4% of them were infected with *T. vaginalis* (26). Sharbatdaran et al. used three methods of direct smear, culture and Pap smears for diagnosis of infection with *T. vaginalis* in Babol (2005) that 18.67%, 18.67% and 25.3% of women were infected respectively (27). 0.9% infection with *T. vaginalis* was observed in 33690 Pap smear specimens in Kermanshah (2005) (28). A survey of women referring to health centers in Tabriz (2006), Yazd (2006), Tehran (2007) showed the percentage of infection with *T. vaginalis* using direct smear and culture was direct smear: 3.46 and culture: 4.56, direct smear: 1.2 and culture: 2.6%, direct smear and culture: 4, direct smear: 22.6, respectively (29–31). In a study that carried out among 300 women referring to health centers of Shahrod (2008) using Pap smear and direct smear methods, only one case of *T. vaginalis* infection was reported (32). A survey of Pap smear samples showed 3.2% of women had a Trichomoniasis infection in the Sari health center (33). Study on women referring to Tehran health center (2009) was achieved using direct smear and culture and obtained result showed the percentage of infection was 2.6 and 3.2, respectively (34). The infection rate of *T. vaginalis* in 160 women suspected of Trichomoniasis in Lorestan was 11.8% and 18.75% using direct smear and culture methods, respectively (35). From 1353 Pap smears specimens in Ahvaz (2010), 1.4% of patients were infected with *T. vaginalis* (36). In two studies in 2010 and 2011, percentage of *T. vaginalis* infection in women with childbearing age and pregnant women in Zanjan was 6.4 and 3.3, respectively (37, 38). PCR-SSCP method was used to test 950 samples from Hamadan and Tehran and obtained results showed fifty samples were positive (39). 3,500 women referring to health centers in Tehran and Kashan were tested using direct smear, culture and PCR methods and 4% of them were infected (40). Patients in Kermanshah, Kashan and rural area of Shahrekord were infected with *T. vaginalis* (Direct smear: 1.5% and culture: 2.1%), (Direct smear and culture: 2%) and (Direct smear: 4%), respectively (41–43). In 2014, women referring to Tehran health centers and Qom were infected with *T. vaginalis* (Culture with urine sample of 5% and vaginal sample of 2.4%, PCR with urine sample of 8.2% and vaginal sample of 8.7%) and (Direct smear: 2.67%, PCR:11.3%), respectively (44, 45) (Table 1).

**Table 1.** Prevalence of *T. vaginalis* in different parts of Iran from 1992 to 2017

**City**	**Method**	**Study population**	**Year**	**Prevalence**
**Sirjan**	Direct smear and culture	500 women referred to health centers	1992	Direct smear: 2.2%, Culture: 2.8%
**Kashan**	Culture	900 women referred to health centers	1993	Culture:2.1%
**Esfahan**	Direct smear and culture	470 women referred to health centers	1995	Direct smear:1.49%, Culture:1.92%
**Tabriz**	Direct smear	469 women referred to health centers	1998	Direct smear: 22.6%
**Yasouj**	Pap smear	1942 Pap smear cytology samples section	1999	Pap smear:1.9%
**Kashan**	Pap smear	1010 Pap smear cytology samples section	1999	Pap smear: 9.9%
**Kashan**	Culture	450 pregnant women referred to health centers	2000	Culture:0.44%
**Zahedan**	Direct smear and culture	597 women referred to health centers	2001	Direct smear:4.5%, culture:5.3%
**Hamedan**	Direct smear	31 women admitted to the psychiatric ward	2001	Direct smear: negative
**Tehran**	Direct smear and culture	***Pregnant women referred to hospital***	2002	Direct smear and culture: 2.9%
**Gorgan**	Direct smear and culture	102 women referred to health centers	2003	Direct smear and culture: 9%
**Hamedan**	Direct smear and culture	400 women referred to health centers	2004	Direct smear:2%, culture:3%
**Orumieh**	Direct smear and culture	420 women referred to health centers	2004	Direct smear:2.4%, culture:2.6%
**Yasouj**	Direct smear and culture and clinical signs	63 women with symptoms of vaginitis	2004	Clinical signs:19.04%, direct smear and culture:42.9%
**Robat Karamu**	Direct smear and culture	500 women referred to health centers	2005	Direct smear and culture:1.4%
**Babol**	Direct smear and culture and Pap smear	150 women with clinical signs	2005	Direct smear:18.67%, culture:18.67%, Pap smear: 25.3%
**Kermanshah**	Pap smear	33690 Pap smear specimens in clinic	2005	Pap smear: 0.9%
**Tabriz**	Direct smear and culture	2630 women referred to health centers	2006	Direct smear:3.46%, Culture: 4.56%
**Yazd**	Direct smear and culture	384 women referred to health centers	2006	Direct smear:1.2%, Culture: 2.6%
**Tehran**	Direct smear and culture	150 women referred to health centers	2007	Direct smear and culture:4%
**Shahrod**	Pap smear and direct smear	300 women referred to health centers	2008	Pap smear and direct smear: Only 1
**Sari**	Pap smear	1832 Pap smear in women referred to health centers	2008	Pap smear: 3.2%
**Tehran**	Direct smear and culture	500 women referred to health centers	2009	Direct smear: 2.6%, Culture: 3.2%
**Lorestan**	Direct smear and culture	160 women suspected of Trichomoniasis	2010	Direct smear:11.8%, Culture:18.75%
**Ahvaz**	Pap smear	1353 Pap smear in women referred to health centers	2010	Pap smear:1.4%
**Zanjan**	Direct smear	328 women referred to health centers	2010	Direct smear: 6.4%
**Zanjan**	Direct smear and culture	1000 pregnant women	2011	Direct smear and culture:3.3%
**Hamadan and Tehran**	PCR-SSCP	950 women referred to health centers	2011	PCR-SSCP: Fifty *T. vaginalis* samples
**Kashan and Tehran**	Direct smear, culture and PCR	3500 women referred to health centers	2011	Direct smear, culture and PCR:4%
**Kermanshah**	Direct smear and culture	600 women referred to health centers	2011–2012	Direct smear1.5%, culture:2.1%
**Kermanshah**	Direct smear and culture	600 women referred to health centers	2012	Direct smear:1.5%, culture:2.1%
**Kermanshah**	Pap smear and direct smear	1100 women referred to health centers	2006–2012	Pap smear and direct smear: 0.63%
**Kashan**	Direct smear and culture	970 women and 235 men referred to health centers	2013	Direct smear and culture :2%
**Shahrekord**	Direct smear	92 rural women	2014	Direct smear:4%
**Tehran**	Culture and PCR	140 women referred to health centers	2014	Culture: urine sample: 5%, vaginal sample: 2.4%, PCR: urine sample: 8.2%, vaginal sample: 8.7%
**Qom**	Direct smear and PCR	300 women referred to health centers	2014	Direct smear: 2.67%, PCR:11.3%
**Ardabil**	Direct smear and culture	904 women referred to health centers	2014	Direct smear: 3.38%, culture: 4.48%
**Hamadan**	Direct smear and culture	1200 women referred to health centers	2015	Direct smear: 0.3%, culture: 0.6%
**Hamadan**	Direct smear and culture	862 women referred to health centers	2015	Direct smear and culture:1.9%

Estimates of prevalence of *T. vaginalis* between populations are different worldwide, but the range from 5–74% in women and 5–29% in men is observed (1). The incidence of Trichomoniasis has increased remarkably especially in developing countries and in populations with high-risk behaviors such as poor sexual activity hygiene and multiple sexual partners. Poverty, socioeconomic status, illiteracy, high risk sexual behaviors, and HIV positive are risk factors for infection of *T. vaginalis* (46–49). In some studies, infection with *T. vaginalis* was more in illiterates than literates (12, 13, 18, 27, 39, 42, 44). Low rate of infection with Trichomoniasis was observed among people who used condoms as a contraceptive method (12, 27, 39, 44), whereas in some studies, Trichomoniasis was not related to prevention methods (13, 14, 42). The reports of Trichomoniasis in Iran are different just the same as other parts of the world. The difference in the prevalence of infection may depend on the selection of population groups, methods and the site of specimen collection. Symptomatic Trichomoniasis is less common in men than women. Biological differences between the two sexes are the cause that women have a higher incidence of infection compared to men (50, 42). Sex hormone is a major factor in different prevalence of Trichomoniasis between both sexes (42). Infection in women can be either asymptomatic or symptomatic, while, it is asymptomatic in men (1–4). Diagnosis of Trichomoniasis on the basis of clinical examinations indicates 88% false negative and 29% false positive results (40). On the basis of some studies, clinical symptoms such as burning and itching were associated with Trichomoniasis (12, 14, 18, 22, 24, 27, 39, 44), while these results were in contrast with other studies (13, 29, 42). Direct smear is the most common method for diagnosis of this infection. After taking samples, they must be tested quickly because the parasite stops moving in a short period and will cause false negative results. The standard method in diagnosis of Trichomoniasis is culture. As this method is sensitive, appropriate conditions including ingredients of culture media, culture temperature, incubation time and the rapid transmission of the parasite after sampling to culture medium are required. Before reporting negative results, negative specimens should be keep up to 7 days for more evaluation. But this method is not used as a routine diagnosis since it wastes much time (44). Typical symptomatic Trichomoniasis in men is cleared spontaneously within 10 days. On the other hand, infection in women can persist for years. Therefore, recognition of carriers is very important for accelerating treatment and decreasing the spread of the disease in control strategies (48, 49). One of the most sensitive diagnostic techniques is polymerase chain reaction (PCR).

For better chances of accurate diagnosis, at least two techniques are needed, such as wet mount microscopy and culture. Although PCR is found to be highly specific and sensitive, it is costly to be used in routine diagnostic laboratories (42, 49, 51). Based on reviewing research conducted in Iran, studies have used at least one detection method (Pap smear, direct smear, culture and PCR) (41–55). Among the articles, only 12 articles had used a detection method while the other remaining articles used at least 2 or 3 detection methods (12–45, 52–55). Research done among various groups of women indicates that the prevalence is different between women admitted to the psychiatric ward and women with symptoms of vaginitis from zero to 42.9%, respectively (21, 26). Results of this systematic review shows the considerable rate of Trichomoniasis in Iran is related to lack of appropriate control programs in different parts of the country. Control of *T. vaginalis* could have considerable public health benefits in controlling both HIV and sexual diseases in women. This approach needs to be conducted in large communities, with particular attention to high-risk groups. Screening or empiric treatment is not only needed in high-risk groups, but also is needed in low-risk groups like pregnant women.

## Conclusion

The present review showed that *T. vaginalis* infection rate is relatively high among the Iranian population. Range of control strategies, including personal hygienic education, simultaneous couple treatment, the sensitivity of diagnostic methods, appropriate preventive tool (condom) in sexual contacts could lead to the disruption of transmission. Using at least two techniques such as culture or PCR in addition to direct smear is recommended for better diagnosis of infection and understanding the actual prevalence of *T. vaginalis*.
